# Natural Variation and Domestication Selection of ZmCKX5 with Root Morphological Traits at the Seedling Stage in Maize

**DOI:** 10.3390/plants10010001

**Published:** 2020-12-22

**Authors:** Houmiao Wang, Hui Sun, Haofeng Xia, Tingting Wu, Pengcheng Li, Chenwu Xu, Zefeng Yang

**Affiliations:** 1 Jiangsu Key Laboratory of Crop Genetics and Physiology/Key Laboratory of Plant Functional Genomics of the Ministry of Education/Jiangsu Key Laboratory of Crop Genomics and Molecular Breeding, Agricultural College of Yangzhou University, Yangzhou 225009, China; houmiaowang@yzu.edu.cn (H.W.); MX120180564@yzu.edu.cn (H.S.); MZ120180903@yzu.edu.cn (H.X.); MZ120201242@yzu.edu.cn (T.W.); pcli@yzu.edu.cn (P.L.); 2 Jiangsu Co-Innovation Center for Modern Production Technology of Grain Crops, Yangzhou University, Yangzhou 225009, China; 3 Joint International Research Laboratory of Agriculture and Agri-Product Safety of Ministry of Education of China, Yangzhou University, Yangzhou 225009, China

**Keywords:** maize, natural variation, *ZmCKX5* gene, root morphological traits

## Abstract

Root system architecture plays a crucial role in water and nutrient acquisition in maize. Cytokinins, which can be irreversibly degraded by the cytokinin oxidase/dehydrogenase (CKX), are important hormones that regulate root development in plants. In this study, *ZmCKX5* was resequenced in 285 inbred lines, 68 landraces, and 32 teosintes to identify the significant variants associated with root traits in maize. Sequence polymorphisms and nucleotide diversity revealed that *ZmCKX5* might be selected during domestication and improvement processes. Marker–trait association analysis in inbred lines identified 12 variants of *ZmCKX5* that were significantly associated with six root traits, including seed root number (SRN), lateral root length (LRL), total root area (RA), root length in 0 to 0.5 mm diameter class (RL005), total root volume (RV), and total root length (TRL). SNP-1195 explained the most (6.01%) phenotypic variation of SRN, and the frequency of this allele G increased from 6.25% and 1.47% in teosintes and landraces, respectively, to 17.39% in inbred lines. Another significant variant, SNP-1406, with a pleiotropic effect, is strongly associated with five root traits, with the frequency of T allele increased from 25.00% and 23.73% in teosintes and landraces, respectively, to 35.00% in inbred lines. These results indicate that *ZmCKX5* may be involved in the development of the maize root system and that the significant variants can be used to develop functional markers to accelerate the improvement in the maize root system.

## 1. Introduction

The root system architecture (RSA) is associated with plants’ ability to absorb water and nutrients from the soil and resist various abiotic stresses in many crops. Varieties with a larger root system are excellent candidates to obtain desired traits, such as faster growth, higher yield, and better abiotic stress tolerance [1]. In the past decades, breeding new varieties is the driving force to achieve higher yields in maize [2]. However, direct selection for optimal RSA is not routine in these maize breeding programs. The potential of root traits for maize improvement remains largely unexploited [3]. Identified gene and natural variation of root growth could help to breed new maize varieties with root traits suitable for diverse environmental conditions.

The root system of maize is composed of the embryonic and postembryonic root system. The embryonic root system includes the primary root (radicle) that is formed at the basal pole of the embryo and a variable number of seminal roots (seed roots) that are laid down at the scutellar node. The postembryonic root system consists of shoot-borne roots (nodal roots or adventitious roots) that are formed at consecutive shoot nodes and lateral roots that are initiated in the pericycle of all roots [4]. These structurally and functionally diverse root types contribute to the complexity of root morphological traits in maize. Root system architecture made up of structural features, such as root length, number, diameter, total area and volume, and length of lateral roots, exhibits great plasticity in response to environmental changes and could be critical to the growth and development of maize [5,6]. Root system development is mediated by various plant endogenous hormones [7], among which auxin and cytokinin play key roles in root development [8]. Cytokinins are involved in the development, morphogenesis, and many other physiological processes of plants. Cytokinins can regulate the elongation of the primary root and inhibit lateral root initiation in Arabidopsis [9]. Arabidopsis mutants with reduced cytokinin content display increased root branching [10,11]. So it is essential to maintain the homeostasis of cytokinins in tissues, cells, and organelles [12]. Cytokinin oxidase/dehydrogenase (CKX) is the only enzyme known to be able to degrade cytokinins irreversibly in active plant cells [13]. The CKX is a multi-gene family in plant genomes. In Arabidopsis, seven CKX genes have been identified and designated *AtCKX1* to *AtCKX7*. Overexpressing *AtCKX1* to *AtCKX4* could increase root length [10,14]. A total of eleven CKX genes have been detected in the genome of rice (*OsCKX1* to *OsCKX11*). Up to now, only *OsCKX2* and *OsCKX4* have been studied in detail. Downregulating *OsCKX2* can increase the tiller number and grain weight, leading to enhanced growth and productivity in rice [15]. A rice dominant mutant root enhancer1 (ren1-D) was observed to exhibit a more robust root system and increased crown root number. Molecular and genetic analyses revealed that these phenotypes are caused by the activation of a cytokinin oxidase/dehydrogenase (CKX) family gene, *OsCKX4* [8]. There are thirteen *ZmCKX* genes in the maize genome, *ZmCKX1* to *ZmCKX12*, and *ZmCKX4b*. Studies have been conducted on the subcellular localization [16] and biochemical characteristics [17] of the *ZmCKX* genes. However, the role of ZmCKX genes in maize root development has not yet been elucidated.

Association analysis is an effective way to analyze the genetic mechanism of complex traits. This research option has been applied to investigate root development in maize [18,19,20,21]. To investigate the natural variation of *ZmCKX* genes, single-nucleotide polymorphism (SNP) in ten CKX genes were filtered from a genotyping-by-sequencing (GBS) dataset in 285 inbred lines. A candidate gene-based association analysis was conducted between *ZmCKX* genes and root traits. Our results showed that *ZmCKX5* were significantly associated with five root traits at the seedling stage (Table S1). We, therefore, re-sequenced the *ZmCKX5* in 285 inbred lines further, 68 landraces, and 32 teosintes, and aimed to (1) examine nucleotide diversity of *ZmCKX5* in maize inbred lines, landraces, and teosintes, (2) identify favorable alleles and haplotypes within *ZmCKX5* that are associated with root morphology, and (3) explore the role of *ZmCKX5* in the domestication and improvement processes of maize. 

## 2. Materials and Methods

### 2.1. Plant Materials and Experimental Design

In this study, a total of 385 lines, including 285 maize inbred lines, 68 landraces, and 32 teosintes (Table S2) from a wide variety of sources, were used. Seeds with a similar appearance to the 285 inbred lines were sterilized in 10% H_2_O_2_ solution for 20 min and then rinsed with distilled water twice. The seeds were soaked in saturated CaSO_4_ solution for 6 h, then germinated at 28 ℃ for two days in a dark environment at 80% relative humidity. Eight seeds of each line were selected and vertically rolled in a double layer of brown germination roll paper (Anchor Paper Company, St Paul, MN, USA). A completely randomized design with two replicates was used. The paper rolls were placed in black incubators and cultured with nutrient solution. The maize seedlings were grown with natural lighting, at 30/26 °C (light/darkness), and 40–70% relative humidity. The composition of the nutrient solution was as follows: 2.0 mmol L^−1^ Ca(NO_3_)_2_∙4H_2_O, 0.75 mmol L^−1^ K_2_SO_4_, 0.65 mmol L^−1^ MgSO_4_∙7H_2_O, 0.25 mmol L^−1^ KH_2_PO_4_, 0.1 mmol L^−1^ KCl, 0.1 mmol L^−1^ EDTA-FeNa, 1 × 10^−3^ mmol L^−1^ ZnSO_4_∙7H_2_O, 1 × 10^−3^ mmol L^−1^ MnSO_4_∙H_2_O, 1 × 10^−3^ mmol L^−1^ H_3_BO_3_, 1 × 10^−4^ mmol L^−1^ CuSO_4_∙5H_2_O, 5 × 10^−6^ mmol L^−1^ (NH_4_)_6_Mo_7_O_24_∙4H_2_O, adjusted the pH of nutrient solution to 6.0 ± 0.05 with NaOH solution [22]. The nutrient solution was renewed every 3 days, aerated by a pump, and the position of the seedbed was changed when the nutrient solution was renewed. The plants were harvested 14 days after germination, and the root number and length of primary and seminal root were measured. The root system was scanned to obtain high-resolution images and analyzed by WinPHIZO software (V2013e, Regent Instruments, Quebec, Canada) (Figure S1) [23]. A total of 12 root traits was measured: primary root length (PRL), seminal root length (SRL), root surface area (RA), root volume (RV), total root length (TRL), lateral root length (LRL), seminal root numbers (SRN), root length between 0 mm and 0.5 mm in diameter (RL005), average root diameter (ARD), root length between 0.5 mm and 1.0 mm in diameter (RL0510), root length between 1.0 mm and 1.5 mm in diameter (RL1015), and root length greater than 1.5 mm in diameter (RL15) (Figure S2).

### 2.2. DNA Isolation, ZmCKX5 Re-Sequencing, and Analysis

The cetyltrimethylammonium bromide (CTAB) method was used to extract genomic DNA from young leaves of inbred lines, landraces, and teosintes [24]. A total of 1521 genes, including *ZmCKX5,* were captured on the NimbleGen platform and sequenced by BGI Life Tech Co. [25]. The genomic sequence of *ZmCKX5* (GRMZM2G325612) from the B73 inbred line (AGPv3.31) was used as a reference for target sequence capture following the manufacturer’s protocols (Roche/NimbleGen) [25]. DNA was sheared by sonication, and adaptors were ligated to the resulting fragments. Extracted DNA with the desired size was amplified by PCR, purified, and hybridized to the capture array at 42.0 °C using the manufacturer’s buffer. The array was washed twice at 47.5 °C and three more times at room temperature. The resulting fragments were purified with the DNA Clean & Concentrator-25 Kit (Zymo Research) and Bioanalyzer (Agilent) and subjected to DNA sequencing on the Illumina platform (Table S3). After removing adapters, low-quality reads, the clean reads were mapped to the genome sequence of B73 (AGPv3.31) by Burrow-Wheeler Aligner (BWA) with the settings ‘mem -t 4 -k 32 -M’ [26]; variant calling and gene sequences converting were performed for all samples using the GATK 4.0 [27]. Multiple sequence alignment of *ZmCKX5* was performed using MAFFT software [28]. The aligned sequences were further edited manually to correct obvious mismatches by BioEdit software [29]. The gene regions of 5′-untranslated region (UTR), 3′-UTR, coding regions, and introns were annotated with B73 reference (AGPv3.31). DNASP6.0 software [30] was used for sequence polymorphism, genetic diversity analysis, and neutral evolution test. Nucleotide sequence polymorphism of 5′-UTR, 3′-UTR, coding regions, and introns were estimated using π and θ, π represents the average number of base differences of any two different sequences in the nucleotide sequence, and θ is derived from the total number of segregating sites and corrected for sampling size [31]. The neutrality test was conducted with Tajima’s D [32], Fu and Li’s D*, and Fu and Li’s F* [33] in the DNASP6.0 software. The linkage disequilibrium (LD) between any two polymorphic sites was estimated using TASSEL5.0 software [34], and *r*^2^ was used to measure the LD level.

### 2.3. Association Analysis between ZmCKX5 and Root Traits

The association between variants of *ZmCKX5* and root traits was performed by TASSEL5.0 with mixed linear models (MLM). To reduce the false positive error, the top five principal components (PCs) and kinship (K) were used to control for population structure and kinship. A total of 559 *ZmCKX5*-based markers with a minor allele frequency (MAF) ≥ 0.05 were selected for association analysis in 285 inbred lines. The *p*-value thresholds were 0.00179 using the bonferroni correction (1/559, −log_10_(*p*) > 2.75). 

## 3. Results

### 3.1. Nucleotide Diversity of ZmCKX5 in Inbred Lines, Landraces, and Teosintes

The genomic sequence of *ZmCKX5* from B73 (RefGen_v3) was used as a reference, and the multiple sequence alignment was performed on all *ZmCKX5* sequences obtained from plant individuals. A total of 6574 bp genomic regions of *ZmCKX5* were sequenced, covering 1562 bp of the upstream (promoter) region, 63 bp of the 5′ untranslated region (UTR), 1818 bp of 3 exons, 2217 bp of 2 introns, 368 bp of 3′UTR, and 544 bp of the downstream region (Table 1). Among these regions, a total of 559 variations were identified, including 446 single-nucleotide polymorphisms (SNPs) and 113 insertion-deletion mutations (Indels). On average, SNPs were found every 14.74 bp, while the Indels were found every 58.18 bp, and the Indels have an average length of 3.36 bp. The highest frequencies of SNPs and Indels were both found in the 3’UTR (1 per 4.61 bp and 1 per 15.38 bp, respectively). The overall nucleotide diversity (π × 1000) of the *ZmCKX5* was calculated to be 12.4 by using DNASP6.0 software. Among different regions of *ZmCKX5*, the nucleotide diversity in the non-coding regions is relatively higher, with the π × 1000 value of 27.7 in the 3′UTR, while the lowest π × 1000 value (3.75) was observed on the first exon (Table 1). To further evaluate whether the gene was selected in the process of maize evolution, the sequences of ZmCKX5 from inbred lines, landraces, and teosintes were tested by the neutral test, including Tajima’s D test and Fu and Li’s test. The Tajima’s D values of all regions were significantly less than 0, and the Fu and Li’s values of all regions significantly less than 0 except for the upstream region. These results indicate that *ZmCKX5* may have been under purifying selection during the maize domestication.

### 3.2. Nucleotide Diversity of ZmCKX5 among Different Populations

**Figure 1:**
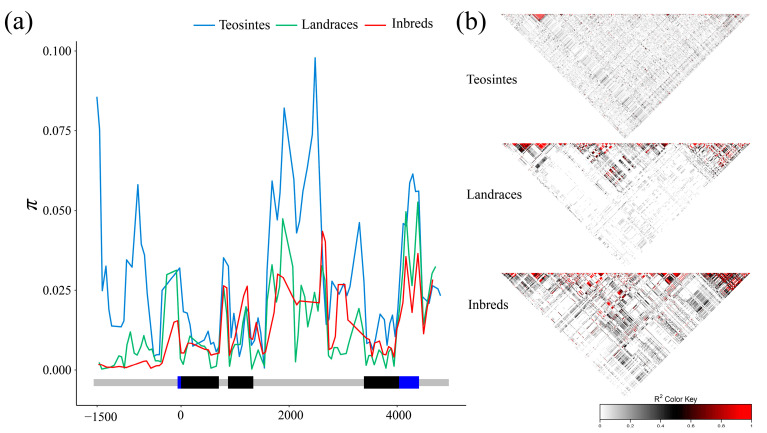
Nucleotide diversity of inbred lines, landrace, and teosintes. (**a**) Nucleotide diversity (π) of inbred lines, landraces, and teosintes. π was calculated using the sliding windows method with a window size of 100 bp and a step length of 25 bp; (**b**) linkage disequilibrium (LD) model of *ZmCKX5* gene.

The sequence conservation (C) and nucleotide diversity (π × 1000) were compared among the three populations. The C and π × 1000 values were 0.729 and 12.40, respectively (Table 2). Inbred lines and landraces showed higher conservation (C_I_ = 0.828; C_L_ = 0.838; C_T_ = 0.779) and lower diversity (π × 1000_I_ = 10.99; π × 1000_L_ = 14.31; π × 1000_T_ = 28.47) than teosintes. Nucleotide diversity was calculated in different regions of *ZmCKX5* among the three populations. The most significant divergence was observed in the upstream region between inbred lines and teosintes, and the highest nucleotide diversity was observed on the second intron in teosintes (Figure 1a). Linkage disequilibrium (LD) analysis showed that the LD blocks of *ZmCKX5* increased from teosintes to landraces and inbred lines (Figure 1b). This result suggests that *ZmCKX5* has been under selection during the domestication process of maize. The Tajima’s D and Fu and Li’s tests were applied on the *ZmCKX5* locus among the three populations. The only significant value was observed for Fu and Li’s D* in inbred lines (Table 2).

### 3.3. Association Analysis of Root Traits with ZmCKX5

**Figure 2:**
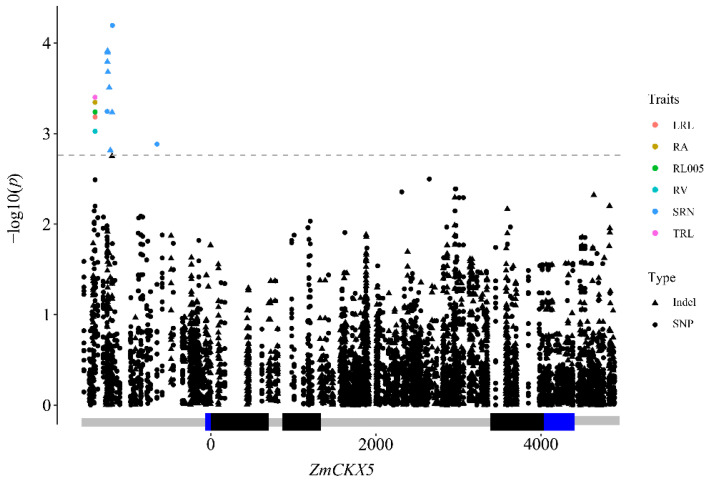
Association analysis between *ZmCKX5* and root traits. Triangles and dots represent InDels and SNPs, respectively. Abbreviations for traits are as follows: LRL, lateral root length; RA, root surface area; RL005, root length between 0 mm and 0.5 mm in diameter (RL005); RV, root volume; SRN, seminal root number; TRL, total root length.

The mixed linear model (PCA + Kinship) was applied to identify the association of root traits with nucleotide polymorphism of *ZmCKX5* in 285 inbred lines. A total of 559 variants with a minor allele frequency (MAF) > 0.05 were included for a trait–marker association analysis. A total of 12 markers (5 SNPs and 7 Indels) were significantly associated (−log_10_(*P*) > 2.75) with at least one root trait (Table 3), including SRN, TRL, RA, RV, RL005, and LRL. The 12 variants were all located in the upstream region of *ZmCKX5* and could explain 3.68–6.01% of the phenotypic variations (Figure 2). 

**Figure 3:**
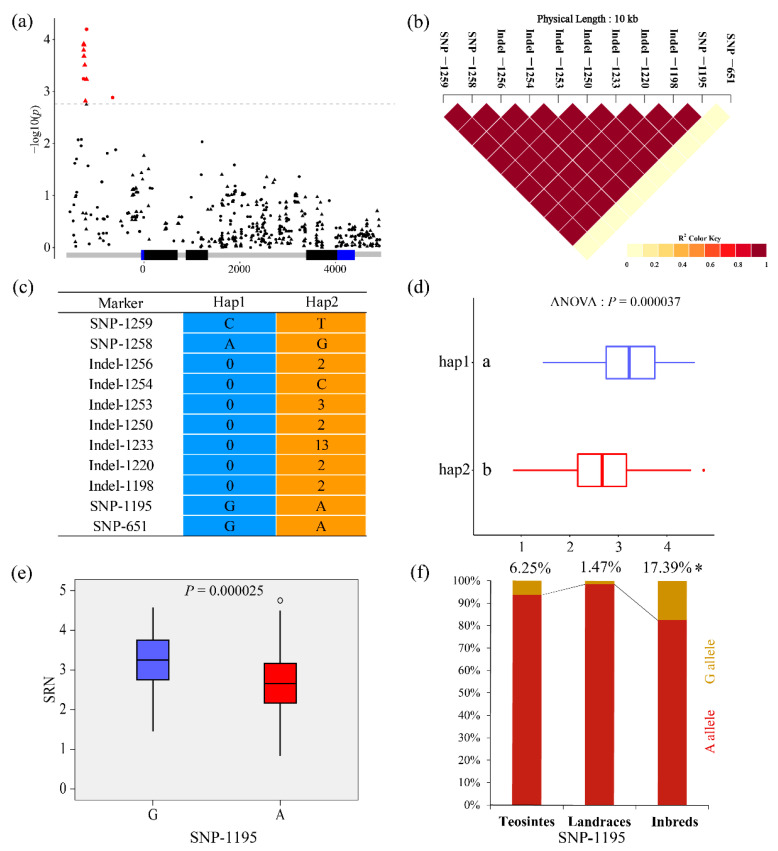
The natural variation of *ZmCKX5* was significantly associated with the seed root number (SRN). (**a**) Trait–marker association between *ZmCKX5* and SRN; (**b**) LD heatmap of 11 variants significantly associated with SRN; (**c**) Haplotypes of *ZmCKX5* among natural variations in inbred lines; (**d**) Comparison of seminal root number between different haplotypes; (**e**) Comparison of seminal root number between different alleles of single-nucleotide polymorphism (SNP)-1195; (**f**) The allele frequency of SNP-1195 in teosintes, landrace, and inbred lines. * indicated a significance different in allele frequency between different group at *p* < 0.05 level by chi-square test.

Among the 12 significant variants, 11 were associated with SRN (Figure 3a), including 4 SNPs and 7 Indels, of which all showed strong LD except SNP-651 (Figure 3b). The 285 inbred lines can be divided into two major haplotypes (Figure 3c) according to these 11 significant variants. The SRN was compared between the two haplotypes, and a significant difference (*P* = 3.7 × 10^−5^) was observed. SNP-1195 contributed the most phenotypic variation (*r*^2^ = 6.01%) to SRN, and the lines carrying the G allele had a significantly greater SRN (*P* = 2.5 × 10^−5^) than those carrying the A allele (Figure 3e). We analyzed the allele frequency of SNP-1195 in inbred lines, landraces, and teosintes and found that the allele frequencies in landraces (1.47%) and teosintes (6.25%) were lower than that in inbred lines (17.39%; Figure 3f).

**Figure 4:**
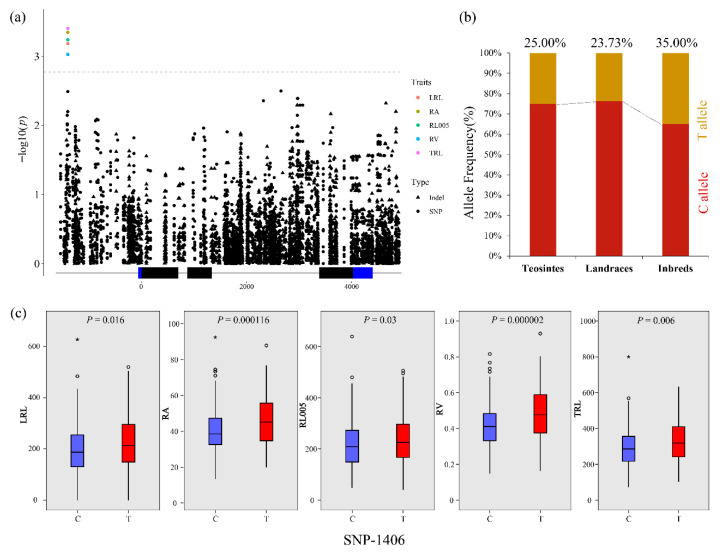
The natural variation of *ZmCKX5* was significantly associated with lateral root length (LRL), total root area (RA), root length in 0 to 0.5 mm diameter class (RL005), total root volume (RV), and total root length (TRL); (**a**) Trait–marker association between *ZmCKX5* and root traits; (**b**) The allele frequency of SNP-1406 in teosintes, landrace, and inbred lines; (**c**) Comparison of LRL, RA, RL005, RV, and TRL between different alleles of SNP-1406. * indicated an individual with extreme value.

SNP-1406 was a pleiotropic variant that was associated with five root traits (Figure 4a) and can explain 3.90%–4.49% phenotypic variations. The lines carrying the T allele had a significantly higher value of root traits than that with the C allele. The allele frequency of SNP-1406 was calculated among the three populations and found that the proportion of individuals carrying the T allele in inbred lines (35.00%) was higher than that in landraces (23.73%) and teosintes (25.00%; Figure 4b).

## 4. Discussion

For a long time, cytokinins have been considered to be involved in several aspects of plant growth and development, such as root development, shoot meristems, leaf senescence, and grain number. The cytokinins are positive shoot growth regulators and negative root growth regulators involved in controlling both shoot architecture and root system architecture [10,35]. Cytokinin oxidase/dehydrogenase catalyzes the irreversible degradation of cytokinin. Manipulation of CKXs showed clear impacts on root development in Arabidopsis, rice, barely, Lotus japonicas, and chickpea [8,10,14,36,37,38]. Collectively, several previous studies revealed that the *CKX* genes were important genetic targets for root improvement and crop yield [35]. Previous studies identified 13 CKX members in maize, but the function of most *CKX* genes is unclear. In this study, we applied gene-based association analysis found that ZmCKX5 may be involved in the development of the maize root system, and significant variants were identified that could be used to develop functional markers for improvement in the maize root system.

Gene-based association analysis is a powerful tool to identify causal variants between gene and target traits [39]. More than 30 genes in maize have been reported by this method. In this study, a total of 12 variants in the upstream region of ZmCKX5 showed significant associations with maize root traits at the seedling stage. A collection of studies has shown that variations in the genes upstream may affect the gene expression and further lead to plant phenotypic alterations. For example, Yang et al. [40] revealed that the insertion of a CACTA-like transposon in the upstream promoter region of *ZmCCT10* in maize disrupts *ZmCCT10* expression and attenuates photoperiod sensitivity under long-day environments. Liang et al. [41] identified an SNP (SNP-1245) in the promoter region of *ZEA CENTRORADIALIS 8* (*ZCN8*) that is strongly correlated with flowering time. The individual carrying the early-flowering SNP-1245A allele showed higher *ZCN8* expression than those carrying the late-flowering SNP-1245G allele. It is worth noting that the promoter sequences in this study were 1500 bp, and some important variations were located more than 2000 bp from the start codon, such as a CACTA-like transposable element ~2.5 kb upstream of ZmCCT [40] and a Hopscotch element ~60 kb upstream of tb1 [42]. There may be important variations far from the start codon of CKX5 (more than 2000kb). It is hypothesized that the identified variants may alter gene expression of CKX5 to regulate the content of cytokinin. Further, we checked the expression patterns of CKXs. Although ZmCKX5 showed a low expression level in 11 CKX genes, it was mainly expressed in primary root, shoot tip, and seed (Table S4). The expression of CKX5 was strongly induced by drought stress, and the expression level could be increased by 8-fold than well-water condition. A study in chickpea found that root-specific expression of CaCKX6 led to a significant increase in lateral root number, root biomass, drought tolerance, and yield [37]. Root engineering with root-specific expressing AtCKX1 and AtCKX2 in Barley generated transgenic barley plants with enhanced cytokinin degradation, which display a larger root system and improved drought tolerance [38]. Taken together, variations in CKX5 may have the potential to develop maize tolerance cultivars to water deficiency by improving root performance. RSA is shaped by multiple traits and developmental processes in a systematic way. Variable correlations between different root traits have been observed in different studies. In hydroponics, the root number and root length were correlated in the same root type with high coefficients (*r* = 0.47–0.65) [43], and crown root diameter was positively correlated with crown root number in the field (*r* = 0.50) [44]. Here, a high correlation (*r* = 0.19–0.97; Table S5) between SRN, TRL, LRL, RL005, RV, and RA was observed. And a pleiotropic variant SNP-1406 was associated with five root traits. These results indicated that these root traits shared a similar regulation mechanism. We also compared our results with the other root GWAS studies both in hydroponics and field, but ZmCKX5 was not picked up by previous GWAS [18,19]. This indicates the complexity of the root regulation mechanism to some extent. Ultimately, multiple traits should be modified in a systematic way to optimize root performance. 

In conclusion, ZmCKX5 was resequenced in 285 inbred lines, 68 landraces, and 32 teosintes to identify the significant variants associated with root traits in maize. Sequence polymorphisms and nucleotide diversity revealed that ZmCKX5 might be selected during domestication and improvement processes. Marker–trait association analysis in inbred lines identified 12 variants of ZmCKX5 that were significantly associated with six root traits. The frequency of the increased allele of significant SNP-1195 and SNP-1406 increased during the maize domestication and improvement processes. The identified significant variants and elite haplotype could be used to improve root traits by molecular breeding.

## Figures and Tables

**Table 1:** Parameters for the sequence variants of *ZmCKX5.*

Parameter	Upstream	5′-UTR	Exon1	Exon2	Exon3	Intron1	Intron2	3′-UTR	Downstream	Entire Region
Total length of amplicons(bp)	1561	64	700	468	650	169	2048	369	545	6574
Number of all of the sequence variants	17	9	55	42	46	28	233	104	32	559
Frequency of all of the sequence variants	0.011	0.141	0.079	0.09	0.071	0.166	0.114	0.282	0.059	0.09
Number of nucleotides substitutions (bp)	10	8	47	35	43	25	180	80	22	446
Frequency of polymorphic sites per bp	0.006	0.125	0.067	0.075	0.066	0.148	0.088	0.217	0.04	0.07
Number of Indels	7	1	8	7	3	3	53	24	10	113
Number of Indel sites	18	1	54	23	15	10	182	49	34	380
Average Indel length Frequency of Indels per bp	0.004	0.016	0.011	0.015	0.005	0.018	0.026	0.065	0.018	0.020
π × 1000	25.13	6.26	3.75	7.77	5.2	25.57	15.23	27.7	19.09	12.4
θ × 1000	54.29	37.32	19.5	16.34	12.24	45.8	35.28	46.55	23.71	26.8
Tajima’s D	−1.199	−1.727	−2.269 **	−1.414	−1.591	−1.148	−1.701	−1.174	−0.507	−1.634
Fu and Li’s D	0.61	−3.281 **	−5.988 **	−4.651 **	−4.480 **	−6.687 **	−7.126 **	−6.995 **	−4.292 **	−8.305 **
Fu and Li’s F	−0.097	−3.271 **	−5.114 **	−3.891 **	−3.811 **	−5.243 **	−5.010 **	−4.925 **	−3.246 **	−5.397 **

π represents the average number of base differences of any two different sequences in the nucleo-tide sequence, θ is derived from the total number of segregating sites and corrected for sampling size; UTR indicated untranslated region; * means a significant difference at 0.05 levels; ** means a significant difference at 0.01 levels.

**Table 2:** *ZmCKX5* genetic diversity analysis and neutral test between teosintes, landraces, and inbred lines.

Population	Hd	Dens.	C	π × 1000	θ × 1000	Tajima‘s D	D	F
Teosintes	1.000	88	0.779	28.47	160.41	−1.338	−1.965	−1.901
Landraces	1.000	55	0.838	14.31	78.72	−0.872	−1.662	−1.527
Inbreds	0.965	35	0.828	10.99	37.96	−0.236	−2.671 *	−1.548
All	0.974	68	0.729	12.40	26.79	−1.634	−8.305 **	−5.397 **

Hd represents haplotype diversity, Dens. Denotes the number of single nucleotide polymorphisms (SNP) per 1000 bp, C represents sequence conservation, *π* represents the average number of base differences of any two different sequences in the nucleotide sequence, *θ* is derived from the total number of segregating sites and corrected for sampling size, and D and F represent Fu and Li’s Dand F. * indicates a statistical significance at *p* < 0.05 level, ** indicates a statistical significance at *p* < 0.01 level.

**Table 3:** Significant markers of *ZmCKX5* associated with root traits.

Traits	Marker	Alleles	*p*-Value	−log_10_(*p*)	*r*^2^ (%)	Region
TRL	SNP-1406	T/C	0.000398	3.40	4.49	Upstream
SRN	SNP-1259	T/C	0.000571	3.24	4.53	Upstream
SRN	SNP-1258	G/A	0.000571	3.24	4.53	Upstream
SRN	Indel-1256	AC/--	0.000161	3.79	5.15	Upstream
SRN	Indel-1254	C/-	0.000122	3.91	5.36	Upstream
SRN	Indel-1253	TCA/---	0.000127	3.90	5.31	Upstream
SRN	Indel-1250	CC/--	0.000209	3.68	4.96	Upstream
SRN	Indel-1233	AAGTGTTAGACTT/-------------	0.000311	3.51	4.70	Upstream
SRN	Indel-1220	TT/--	0.001530	2.82	3.81	Upstream
SRN	Indel-1198	CA/--	0.000582	3.24	4.53	Upstream
SRN	SNP-1195	A/G	0.000064	4.19	6.01	Upstream
SRN	SNP-651	A/G	0.001310	2.88	3.68	Upstream
RV	SNP-1406	T/C	0.000942	3.03	3.90	Upstream
RL005	SNP-1406	T/C	0.000578	3.24	4.18	Upstream
RA	SNP-1406	T/C	0.000452	3.34	4.43	Upstream
LRL	SNP-1406	T/C	0.000656	3.18	4.18	Upstream

## Data Availability

The sequencing data were deposited in the NCBI Short Read Archive database with the accession number SUB8457389. The raw images of roots can be down-loaded by https://pan.baidu.com/s/1S_kbL25xR2fb4mI3f__8Gw.

## Supplementary Materials

The following are available online at https://www.mdpi.com/2223-7747/10/1/1/s1, Figure S1: Process for measuring maize root traits in the seedling stage; Figure S2: Distribution of root and shoot traits in maize inbred lines; Table S1: Significant variants of ZmCKX associated with root traits detected by gene-based association analysis; Table S2: The list of 285 inbred lines, 68 landraces, and 32 teosintes used in this study; Table S3: Summary of the sequence data for all test lines; Table S4: CKX genes expression patterns extracted from qTeller; Table S5: Correlation analysis among 12 root traits.
