# Supplementary material for: Natural Variation and Domestication Selection of ZmCKX5 with Root Morphological Traits at the Seedling Stage in Maize
Source: Plants (Basel). 2020 Dec 22;10(1):1. doi: 10.3390/plants10010001 (PMC7830956; doi:10.3390/plants10010001)
Supplement: Supplementary file 1 [file plants-10-00001-s001.zip › Supplementary Materials-proof/Supplementary Materials 1.docx]

Supplementary Materials

Natural variation and domestication selection of ZmCKX5 with root morphological traits at the seedling stage in maize

Houmiao Wang ^1,2#^, Hui Sun ^1#^, Haofeng Xia ^1^, Pengcheng Li ^1,2^, Chenwu Xu ^1,2,3*^, Zefeng Yang ^1,2,3*^

^1^ Jiangsu Key Laboratory of Crop Genetics and Physiology/ Key Laboratory of Plant Functional Genomics of the Ministry of Education/ Jiangsu Key Laboratory of Crop Genomics and Molecular Breeding, Agricultural College of Yangzhou University, Yangzhou 225009, China

^2^ Jiangsu Co-Innovation Center for Modern Production Technology of Grain Crops, Yangzhou University, Yangzhou 225009, China

^3^ Joint International Research Laboratory of Agriculture and Agri-Product Safety of Ministry of Education of China, Yangzhou University

***** Correspondence: [**cwxu@yzu.edu.cn**](mailto:cwxu@yzu.edu.cn)**;** [zfyang@yzu.edu.cn](mailto:zfyang@yzu.edu.cn); Tel.: 86-0514-87979358

^#^ These authors have contributed equally to this work

**Supplementary Materials**

**Figure S1.** Process for measuring maize root traits in seedling stage

**Figure S2.** Distribution of root and shoot traits in maize inbred lines

**Table S1.** Significant variants of ZmCKX associated with root traits detected by gene-based association analysis

**Table S3.** Summary of the sequence data for all test lines

**Table S5.** Correlation analysis among 12 root traits


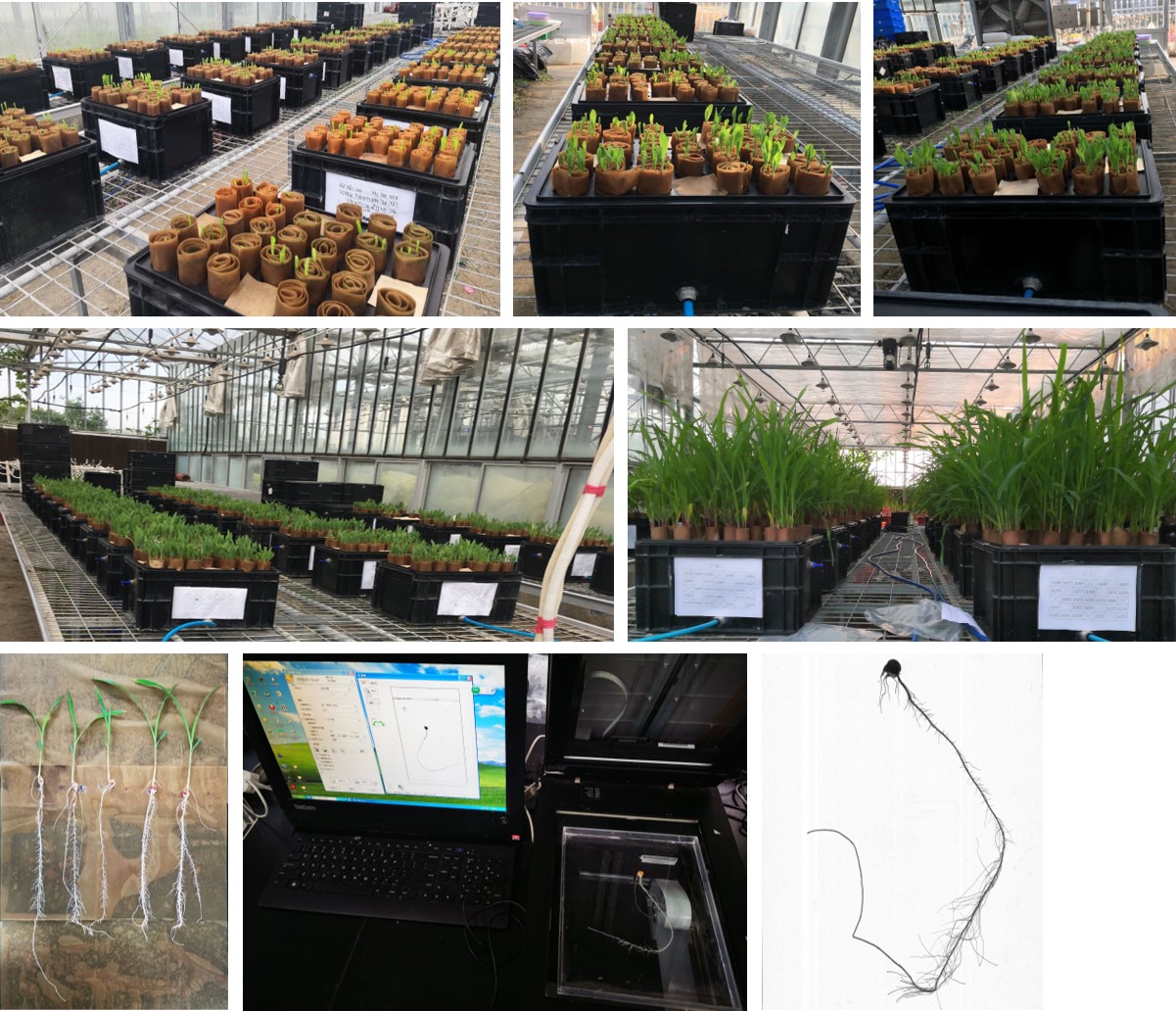


**Figure S1.** Process for measuring maize root traits in seedling stage


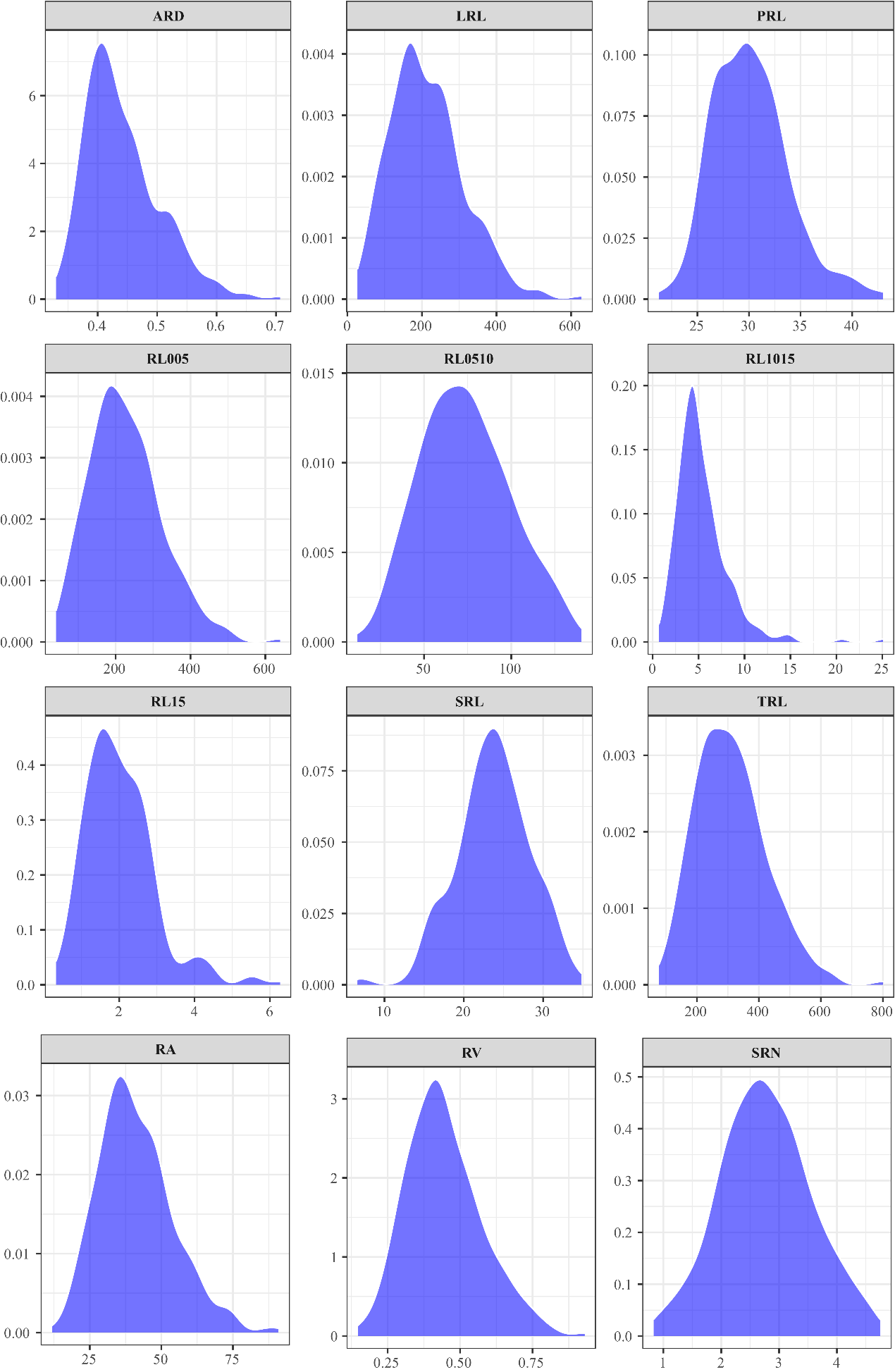


**Figure S2.** Distribution of root and shoot traits in maize inbred lines

**Table S1.** Significant variants of ZmCKX associated with root traits detected by gene-based association analysis

| Gene Name | Traits | Marker | Alleles | *p* value | -lg(p) | r2(%) | Region |
| --- | --- | --- | --- | --- | --- | --- | --- |
| ZmCKX5 | TRL | SNP-1406 | T/C | 0.000398 | 3.40 | 4.49% | Upstream |
| ZmCKX5 | SRN | Indel-1256 | 2/0 | 0.000161 | 3.79 | 5.15% | Upstream |
| ZmCKX5 | SRN | Indel-1254 | C/- | 0.000122 | 3.91 | 5.36% | Upstream |
| ZmCKX5 | SRN | Indel-1253 | 3/0 | 0.000127 | 3.90 | 5.31% | Upstream |
| ZmCKX5 | SRN | Indel-1250 | 2/0 | 0.000209 | 3.68 | 4.96% | Upstream |
| ZmCKX5 | SRN | Indel-1233 | 13/0 | 0.000311 | 3.51 | 4.70% | Upstream |
| ZmCKX5 | SRN | SNP-1195 | A/G | 0.000064 | 4.19 | 6.01% | Upstream |
| ZmCKX4b | ARD | SNP364 | G/C | 0.000300 | 3.52 | 4.17% | Exon1 |
| ZmCKX4b | RS | SNP3504 | A/G | 0.000159 | 3.80 | 9.57% | Intron4 |
| ZmCKX6 | CRN | InDel-1511 | 2/0 | 0.000101 | 4.00 | 4.57% | Upstream |
| ZmCKX6 | SRL | InDel2427 | C/- | 0.000124 | 3.91 | 5.15% | Intron2 |
| ZmCKX7 | CRA | SNP-1446 | T/A | 0.000093 | 4.03 | 5.17% | Upstream |
| ZmCKX12 | SRN | SNP-1707 | T/G | 0.000027 | 4.57 | 6.66% | Upstream |
| ZmCKX12 | SRN | SNP-1722 | A/G | 0.000136 | 3.87 | 5.63% | Upstream |

**Table S3.** Summary of the sequence data for all test lines

|  | Min | Max | Average |
| --- | --- | --- | --- |
| Raw reads | 34,765,308 | 63,349,686 | 51,609,932 |
| Raw bases (Mb) | 5214.8 | 9502.45 | 7741.49 |
| Clean reads | 33,544,414 | 61,198,762 | 50,003,910 |
| Clean bases (Mb) | 4957.78 | 9039.73 | 7369.94 |
| Clean data rate (%) | 94.75 | 95.4 | 95.20 |
| Clean read1 Q20 (%) | 98.35 | 98.45 | 98.41 |
| Clean read2 Q20 (%) | 95.85 | 96.65 | 96.28 |
| Clean read1 Q30 (%) | 95.27 | 95.51 | 95.40 |
| Clean read2 Q30 (%) | 90.15 | 91.83 | 91.05 |
| GC content (%) | 45.96 | 47.66 | 46.45 |

**Table S5.** Correlation analysis among 12 root traits

|  | SRL | RL005 | RL0510 | RL1015 | TRL | LRL | RL15 | ARD | RA | RV | SRN |
| --- | --- | --- | --- | --- | --- | --- | --- | --- | --- | --- | --- |
| PRL | 0.683^**^ | 0.436^**^ | 0.541^**^ | 0.338^**^ | 0.480^**^ | 0.420^**^ | 0.281^**^ | -0.166^**^ | 0.533^**^ | 0.508^**^ | 0.137^*^ |
|  | SRL | 0.621^**^ | 0.646^**^ | 0.314^**^ | 0.663^**^ | 0.599^**^ | 0.323^**^ | -0.364^**^ | 0.666^**^ | 0.557^**^ | 0.158^**^ |
|  |  | RL005 | 0.562^**^ | 0.506^**^ | 0.969^**^ | 0.973^**^ | 0.484^**^ | -0.682^**^ | 0.883^**^ | 0.626^**^ | 0.243^**^ |
|  |  |  | RL0510 | 0.456^**^ | 0.698^**^ | 0.610^**^ | 0.457^**^ | -0.036 | 0.825^**^ | 0.880^**^ | 0.557^**^ |
|  |  |  |  | RL1015 | 0.544^**^ | 0.576^**^ | 0.762^**^ | 0.014 | 0.676^**^ | 0.729^**^ | 0.111 |
|  |  |  |  |  | TRL | 0.963^**^ | 0.529^**^ | -0.579^**^ | 0.939^**^ | 0.733^**^ | 0.334^**^ |
|  |  |  |  |  |  | LRL | 0.536^**^ | -0.625^**^ | 0.902^**^ | 0.683^**^ | 0.193^**^ |
|  |  |  |  |  |  |  | RL15 | 0.024 | 0.659^**^ | 0.711^**^ | 0.126^*^ |
|  |  |  |  |  |  |  |  | ARD | 0-.343^**^ | 0.041 | 0.040 |
|  |  |  |  |  |  |  |  |  | RA | 0.907^**^ | 0.409^**^ |
|  |  |  |  |  |  |  |  |  |  | RV | 0.464^**^ |
|  |  |  |  |  |  |  |  |  |  |  | SRN |
